# The role of retinoic acid in hepatic lipid homeostasis defined by genomic binding and transcriptome profiling

**DOI:** 10.1186/1471-2164-14-575

**Published:** 2013-08-28

**Authors:** Yuqi He, Lei Gong, Yaping Fang, Qi Zhan, Hui-Xin Liu, Yanliu Lu, Grace L Guo, Lois Lehman-McKeeman, Jianwen Fang, Yu-Jui Yvonne Wan

**Affiliations:** 1 Department of Medical Pathology and Laboratory Medicine, University of California, Davis Health Systems, Sacramento 95817, CA, USA; 2 Discovery Toxicology, Bristol-Myers Squibb Company, Princeton 08543, NJ, USA; 3 Applied Bioinformatics Laboratory, University of Kansas, Lawrence, KS, USA; 4 Department of Gastroenterology Hepatology, First Municipal People’s Hospital of Guangzhou, Guangzhou Medical College, Guangzhou 510180, China; 5 Department of Pharmacology and Toxicology, Ernest Mario School of Pharmacy, Rutgers University, Piscataway 08854, NJ, USA; 6 Biometric Research Branch, National Cancer Institute, 9609 Medical Center Dr. Rockville, Rockville 20850, MD, USA

**Keywords:** Nuclear receptor, Retinoids x receptor, Retinoic acid receptor, Farnesnoid x receptor, Peroxisomal proliferator-activated receptor α, Liver x receptor, Pregnane x receptor, Chromatin immunoprecipitation, Sequencing, Microarray

## Abstract

**Background:**

The eyes and skin are obvious retinoid target organs. Vitamin A deficiency causes night blindness and retinoids are widely used to treat acne and psoriasis. However, more than 90% of total body retinol is stored in liver stellate cells. In addition, hepatocytes produce the largest amount of retinol binding protein and cellular retinoic acid binding protein to mobilize retinol from the hepatic storage pool and deliver retinol to its receptors, respectively. Furthermore, hepatocytes express the highest amount of retinoid x receptor alpha (RXRα) among all the cell types. Surprisingly, the function of endogenous retinoids in the liver has received very little attention.

**Results:**

Based on the data generated from chromatin immunoprecipitation followed by sequencing, the global DNA binding of transcription factors including retinoid x receptor α (RXRα) along with its partners i.e. retinoic acid receptor α (RARα), pregnane x receptor (PXR), liver x receptor (LXR), farnesoid x receptor (FXR), and peroxisome proliferator-activated receptor α (PPARα) has been established. Based on the binding, functional annotation illustrated the role of those receptors in regulating hepatic lipid homeostasis. To correlate the DNA binding data with gene expression data, the expression patterns of 576 genes that regulate lipid homeostasis were studied in wild type and liver RXRα-null mice treated with and without RA. The data showed that RA treatment and RXRα-deficiency had opposite effects in regulating lipid homeostasis. A subset of genes (114), which could clearly differentiate the effect of ligand treatment and receptor deficiency, were selected for further functional analysis. The expression data suggested that RA treatment could produce unsaturated fatty acids and induce triglyceride breakdown, bile acid secretion, lipolysis, and retinoids elimination. In contrast, RXRα deficiency might induce the synthesis of saturated fatty acids, triglyceride, cholesterol, bile acids, and retinoids. In addition, DNA binding data indicated extensive cross-talk among RARα, PXR, LXR, FXR, and PPARα in regulating those RA/RXRα-dependent gene expression levels. Moreover, RA reduced serum cholesterol, triglyceride, and bile acid levels in mice.

**Conclusions:**

We have characterized the role of hepatic RA for the first time. Hepatic RA mediated through RXRα and its partners regulates lipid homeostasis.

## Background

The eyes and skin are obvious retinoid target organs. Vitamin A deficiency causes night blindness and retinoids are widely used to treat acne and psoriasis. However, more than 90% of total body retinol (retinylpalmitate, the storage form) is stored in liver stellate cells [1]. In addition, hepatocytes produce the largest amount of retinol binding protein and cellular retinoic acid binding protein to mobilize retinol from the hepatic storage pool and deliver retinol to its receptors, respectively [2]. Furthermore, hepatocytes express the highest amount of retinoid x receptor alpha (RXRα) among all the cell types. Surprisingly, the function of endogenous retinoids in the liver has received very little attention. Thus, the current study aims to identify the bona fide RXRα and RARα targets in the liver.

The broad and complicated roles of retinoids can be explained by the presence of multiple receptors for retinoic acid (RA), the biological active form of retinol. The receptors for RA are retinoic acid receptor (RAR) as well as retinoid x receptor (RXR) [2]. In addition, RXR is essential for many other receptors to function. These receptors for RA belong to a nuclear receptor family whose members are transcriptional factors. Thus, RA exerts its biological effects by regulating gene expression. RXR is unique in that it not only forms homodimers, but also dimerizes with other nuclear receptors, which include receptors for fatty acids (peroxisomal proliferator-activated receptors, PPARs), bile acids (farnesoid x receptor, FXR), oxysterols (liver x receptor, LXR), xenobiotics (pregnane x receptor, PXR, and constitutive androstane receptor, CAR), vitamin D (vitamin D receptor, VDR), and RA (RAR). Hence, most RXR partners participate in regulating lipid homeostasis. Within these heterodimers, RXR can be either a permissive or a silent partner. When RXR serves as a silent partner, the heterodimer does not respond to RA. When it is a permissive (active) partner, RA and the ligand for the heterodimeric partner can both activate the heterodimer. For example, RXR is a permissive partner for PPAR [3]. Similarly, heterodimeric complexes of RXR with LXR [4] or FXR [5] also retain RA responsiveness. Furthermore, retinoids also activate PXR, VDR, and CAR thus are able to thus regulate xenobiotic metabolism and potentially their own oxidation [6-8]. Since most of these receptors are abundantly expressed in the liver, the endogenous RA may regulate many hepatic nuclear receptor-mediated pathways. Therefore, the role of RA in the liver is unpredictable. In order to understand the endogenous function of RA and its receptors, it is crucial to identify RA receptor targets (genes and pathways) genome-wide.

RXRα is highly expressed in the liver [9]. Liver specific RXRα-deficient mice have increased serum triglyceride and cholesterol levels [10,11]. In addition, lack of hepatic RXRα increases sensitivity to alcohol- and non-alcohol-induced steatosis and steatohepatitis [12,13]. Besides regulating lipid metabolism, hepatocyte RXRα also controls xenobiotic [14-16], carbohydrate [17], and amino acid metabolism [17]. These findings indicate that RXRα-mediated signaling has a huge impact on maintaining liver health and in regulating many disease processes.

To understand the global roles of RXRα and RARα at the genomic level, chromatin immunoprecipitation using anti-RXRα and -RARα antibodies followed by sequencing (ChIP-seq) was performed. Since RXRα is an essential partner for other nuclear receptors, we compared ChIP-seq data to RXRα binding locations with locations from previous studies for PXR [18], LXR [19], FXR [20], and PPARα [19]. Meanwhile, the expression levels of the genes responsible for lipid homeostasis were studied in wild type and hepatic RXRα-deficient mouse livers. Both genome-wide DNA-binding and hepatic gene expression data were used to define the role of RA in the liver. Our data uncovered the unknown function of retinoic acid and RXR vs. RAR in the liver. Using different approaches, we showed for the first time that retinoic acid-activated RXRα and RARα have distinct effects. Moreover, the action of retinoic acid in the liver is to regulate lipid homeostasis specifically by reducing serum cholesterol, triglyceride and bile acid levels. The data provided may lead to future development of synthetic retinoid that can target metabolic syndrome or other types of lipid-associated health issues.

## Results

### Genome-wide binding of RXRα, RARα, PXR, LXR, FXR, and PPARα in mouse livers

**Figure 1:**
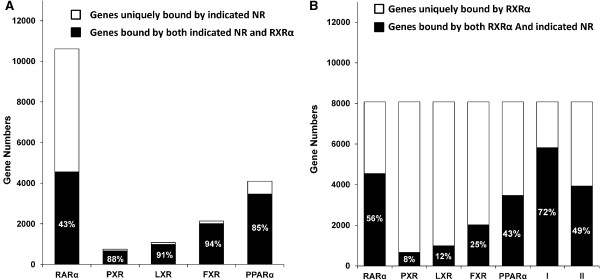
**Histograms showing genome-wide binding of RXRα/nuclear receptor in wild type mouse livers. (A)** The overlapping binding between indicated nuclear receptor (NR) and RXRα. **(B)** The preferential dimerization of RXRα with nuclear receptors. The number and percentage of genes bound by RXRα and indicated nuclear receptors are shown in black. The binding occurs in the same location within a gene. I: the number and percentage of overlapping binding between RXRα and any one of the indicated nuclear receptors. II: the number and percentage of overlapping binding between RXRα and any one of PXR, LXR, FXR, and PPARα.

To understand the global roles of RXRα and RARα at the hepatic genome level, ChIP-seq was performed using anti-RXRα and -RARα antibodies. Single read sequencing yielded 18 and 32 million uniquely mapped reads for RXRα and RARα, respectively. After filtering by including peak scores that were greater than 20 and distance within 10 kb from the transcriptional start site, 17,973 peaks were detected for RXRα and 18,697 peaks for RARα. Since RXRα is an essential partner for other nuclear receptors, we compared ChIP-seq data of RXRα with those of PXR [18], LXR [19], FXR [20], and PPARα [19]. Our data showed that the numbers of peaks, which were commonly bound by RXRα and other nuclear receptors, were 6,577 for RARα, followed by 5,154 for PPARα, 2,846 for FXR, 1,190 for LXR, and 868 for PXR in the mouse liver genome. RARα had less than 50% overlapping bindings with RXRα while PPARα, FXR, LXR, and PXR had over 85% overlapping bindings with RXRα (Figure 1A). These findings suggest that RXRα is indispensable for the function of PPARα, FXR, LXR and PXR in the mouse liver. In contrast, RARα may work in the absence of RXRα. More than 4000 of RARα binding genes were bound by RXRα, and occupied 56% of total RXRα bindings, followed by PPARα (43%), FXR (25%), LXR (12%), and PXR (8%). The well-known lipid regulators including PPARα, FXR, LXR, and PXR bound to almost 50% of RXRα-bound regions suggesting the extensive role of RXRα in regulating lipids.

**Figure 2:**
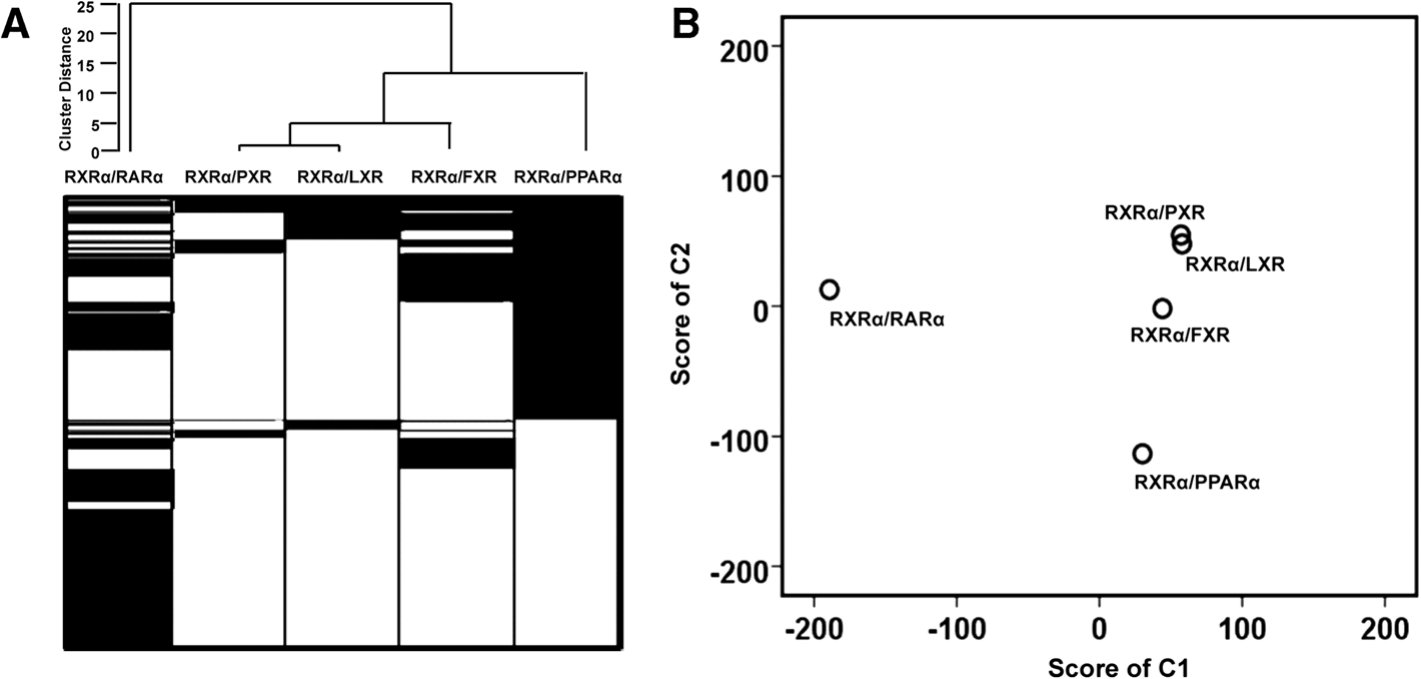
**Genome-wide profiling of RXRα/nuclear receptor binding sites. (A)** A dendogram was generated by cluster analysis to show RXRα/nuclear receptor (NR) binding sites. Each horizontal line represents a binding site. White regions indicate there is no peak called. The lengths of the vertical lines represent the distance between the sets of target genes for the nuclear receptors when clustered. **(B)** A score plot of PCA analysis for RXRα/nuclear receptors binding sites. Scores of component 1 and 2 were obtained from the linear combination of all the nuclear receptors binding sites.

Cluster and principal component analysis (PCA) were performed using the binding data. Overlapping bindings between RXRα and RARα, PPARα, FXR, LXR, and PXR were analyzed. According to the length of the vertical branches (Figure 2A), LXR and PXR were clustered into one basic group. FXR was clustered into a subgroup with LXR and PXR. PPARα, PXR, LXR, and FXR were classified into another group. However, RARα was distant from the others. The same datasets were subjected to PCA analysis. After dimension deduction, two components were picked to describe the global properties of the samples. The two-dimension score plot showed that LXR and PXR had the shortest distance, implying their properties on the genome-wide binding level were most similar (Figure 2B). The distance between FXR and LXR/PXR was shorter than the distances between other receptors and LXR/PXR. RARα was far from all other nuclear receptors. Thus, the information generated from the PCA and cluster analysis was consistent. The difference among the binding profiles of PXR, LXR, FXR, and PPARα were described by component 2 while the difference between the binding profile of RARα and those four nuclear receptors were described by component 1 in the PCA.

### Function analysis of the genes that have overlapping binding sites between RXRα and each of RARα, PXR, LXR, FXR, and PPARα

To understand the potential biological role of genes likely to be targeted by RXRα-heterodimers, DAVID functional annotation was performed. The top ten processes for each of the heterodimers formed by RXRα and other nuclear receptors were investigated in this study (Table 1). Although common pathways among these heterodimers, such as carboxylic acid catabolic process, organic acid catabolic process, and oxidation reduction, appeared in the top 10 pathways, the heterodimers still have their unique biological functions. RXRα-RARα uniquely regulated protein transport, localization, and RNA processing. The heterodimers of RXRα and PXR, LXR, FXR, and PPARα have a great impact on various lipid processing pathways. For example, RXRα-PPARα and RXRα-LXR tend to bind to the genes involved in acylglycerol, glycerol ether, and neutral lipid metabolic processes, which are fatty acid derived. In addition, RXRα-PPARα distinctly binds to the genes that are involved in fatty acid metabolic processes, and RXRα-LXR is more prone to bind to the genes that participate in steroid metabolic process. In addition to steroid metabolic process, RXRα-FXR also tends to bind to genes that dictate lipid transport and regulate steroid and monosaccharide homeostasis. RXRα-PXR targets specific biological processes like regulating pyruvate and carbohydrate biotransformation, but it also has a role in acute-phase response, acute inflammatory response, and response to wounding. Taken together, RXRα-RARα targeted protein and RNA processes while RXRα-PXR/LXR/FXR/PPARα targeted homeostasis regulation of small molecules, which include monosaccharide and lipids.

**Table 1:** Common and unique biological function of genes bound by RXRα coupled with RARα, PXR, LXR, FXR and PPARα

**Pathways**	**RARα**	**PXR**	**LXR**	**FXR**	**PPARα**
Acylglycerol metabolic process			1.9E-07		3.8E-10
Neutral lipid metabolic process			2.7E-07		9.4E-10
Glycerol ether metabolic process			2.7E-07		1E-10
Organic ether metabolic process					4.5E-11
Carboxylic acid catabolic process	6.1E-11	7.3E-07	2.8E-08	1.9E-09	6.1E-11
Organic acid catabolic process	6.1E-11	7.3E-07	2.8E-08	1.9E-09	6.1E-11
Coenzyme metabolic process	9.4E-13			2E-07	1.7E-10
Cofactor metabolic process	1.6E-17	3.0E-06		2.1E-07	2E-11
Fatty acid metabolic process					1.8E-12
Oxidation reduction	1.7E-23	1E-11	1.4E-16	6.1E-14	3.4E-28
Sterol homeostasis			7.2E-08		
Cholesterol homeostasis			7.2E-08		
Lipid homeostasis				4.8E-07	
Lipid transport				2E-07	
Hexose metabolic process				2.1E-07	
Steroid metabolic process			2.6E-11	2E-08	
Monosaccharide metabolic process				1.4E-07	
Pyruvate metabolic process		1.3E-05			
Acute-phase response		3.2E-05			
Cellular carbohydrate biosynthetic process		5.9E-06			
Acute inflammatory response		7.3E-07			
Glucose metabolic process		0.00001			
Response to wounding		3.5E-07			
Electron transport chain	2.7E-10				
ncRNA metabolic process	4.2E-15		1.8E-09		
mRNA processing	6.7E-16				
Translation	7E-16				
RNA processing	8.8E-16				

Numbers in the table represent the *p* value given by DAVID.

### Global profiling of the expression of lipid homeostasis genes in wild type and RXRα KO mice treated with and without RA

**Figure 3:**
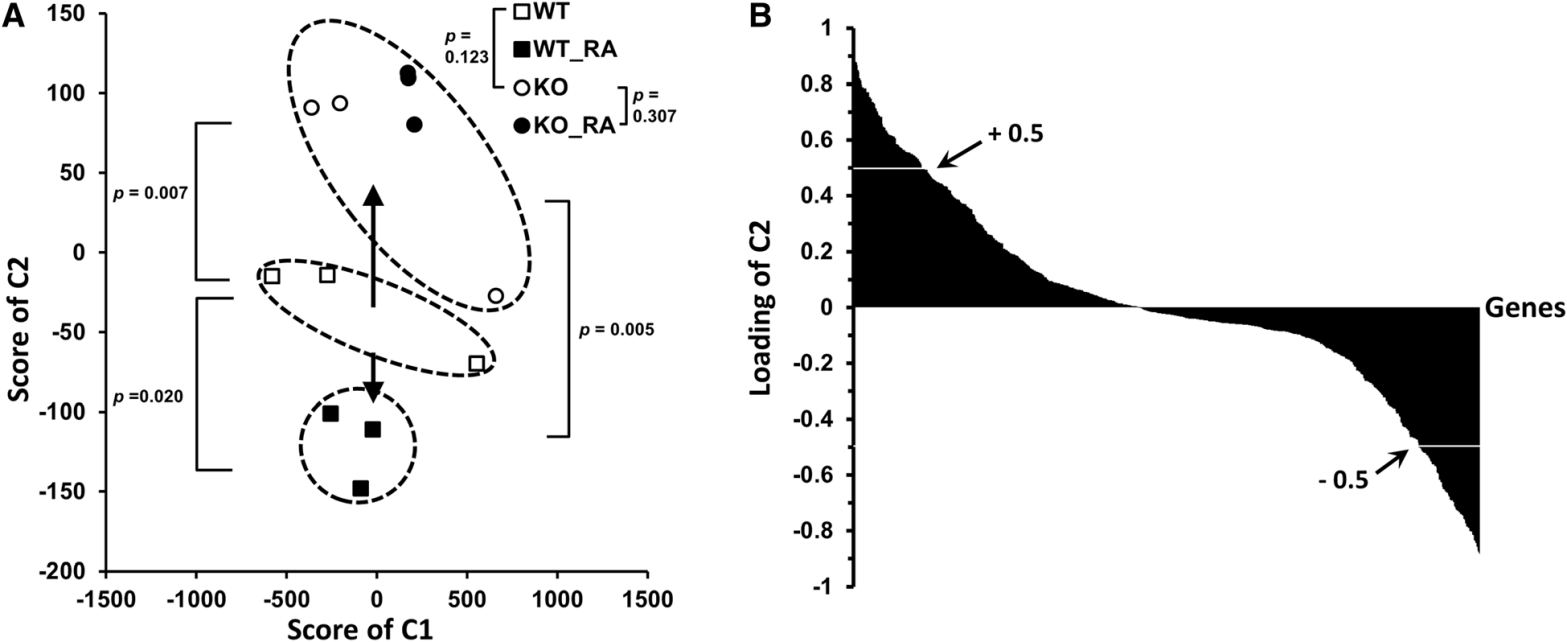
**PCA of the expression level of 579 lipid genes in wild type and hepatic RXRα-KO mice treated with and without RA.** Wild type and hepatic RXRα KO mice were treated with and without RA for 7 days (150 mg/kg diet, n = 3). The expression of 579 genes involved lipid homeostasis was studied. **(A)** Score plot of PCA showing the difference among the groups. Spots within an ellipse belong to the same group. Arrows represent the direction separating groups from the wild type (WT) mice (open square □). There is no significance in the C1component observed across all groups. **(B)** A histogram that shows the loading value of genes on C2. Genes with high loading value (≥ 0.5) on C2 had higher mRNA levels in RXRα KO than wild type livers. In contrast, genes with low loading value (≤ -0.5) on C2 had increased mRNA levels due to RA treatment.

Hepatic RXRα KO mice have elevated serum cholesterol and triglyceride levels [21], and the majority of PXR, LXR, FXR, PPARα-bound genes were also bound by RXRα. We next tested a hypothesis that RA and hepatic RXRα could regulate lipid homeostasis in the mouse liver via RXRα and its heterodimeric partners. The expression levels of the lipid homeostasis genes (579) in the KEGG pathway database were studied in wild type and hepatic RXRα KO mice treated with and without RA. The PCA score plot showed that RA treatment of wild type mice caused a downward shift in C2 from the untreated control group (Figure 3A). In contrast, an upward shift was found due to hepatic RXRα deficiency in comparison to the untreated wild type mice. Thus, RA treatment and RXRα deficiency had opposite effects. In addition, no significant change was noted when RA was used to treat hepatic RXRα KO mice. These findings unequivocally prove that the effects of RA on regulating those lipid homeostasis genes were RXRα dependent. Score plot (Figure 3A) indicates component 2 made a contribution to distinguish groups of control, RA-treated, and RXRα-deficient mice. Thus, 114 out of 579 genes with high loading values (>0.5 or < -0.5) in component 2 were selected for further analysis (Figure 3B). Among them, 55 genes were induced by RA and had decreased expression levels due to RXRα deficiency. The other 59 genes, whose expression levels were suppressed by RA, had increased expression levels due to a lack of RXRα. Thus, the expression levels of those 114 lipid-related genes are ligand (RA)-responsive and receptor (RXRα)-dependent. Based on the known function of those genes described in KEGG and PubMed, the role of those 114 genes was assigned and summarized in Table 2. Remarkably, RA regulated many genes involved in certain pathways. For example, RA decreased the expression of 10 genes in the cholesterol biosynthesis pathway, but did not increase the expression of any other genes in the same pathway. Thus, it is very likely that RA inhibited the biosynthesis of cholesterol in an RXRα-dependent manner. RA also induced the expression of 13 genes in the RA elimination process and yet did not reduce the expression of any gene in the same process. Thus, RA can self-regulate its own level. Furthermore, RA also induced the expression of 9 genes in the biosynthesis of unsaturated fatty acids responsible for anti-inflammation. Since there was no inhibition of gene expression in the same pathway, it is very likely that RA up-regulates the synthesis of unsaturated fatty acids and has an anti-inflammatory role. ChIP-Seq data indicated that most of the genes (87 out of 114) had RXRα binding implying direct gene regulation.

**Table 2:** Biological functions responding to RA treatment and RXRα knockout in wild type mice

	**Gene number (with RXRα bindings)**							
**Biological functions**	**RA induced & RXRα KO repressed**				**RA repressed & RXRα KO induced**			
Lipid droplet growth		1	(0)			2	(2)	
Transportation of bile aicds for bile excretion		1	(0)			0		
Dehydrogenation of saturated fatty acids to unsaturated fatty acids		3	(2)			0		
Biosynthesis of glycerol phosphalipids		4	(3)			1	(1)	
Tryglyceride degration		4	(3)			0		
Biosynthesis of unsaturated fatty acids responsible for aniti-inflammation		9	(7)			0		
Elimination of retinoic acids		13	(11)			0		
Biosynthesis of bile acids		4	(4)			4	(4)	
Biosynthesis of retinoic acids		1	(1)			2	(2)	
Biosynthesis of saturated fatty acids		5	(5)			10	(10)	
Degradation of glycerol phosphalipids		1	(1)			4	(4)	
Degradation of saturated fatty acids		10	(10)			5	(5)	
Elimination of steroid hormones		3	(3)			5	(5)	
Fat digestion and absorption		3	(3)			7	(7)	
Lipid droplet breakdown (fat mobilization)		1	(1)			1	(1)	
Recycle of bile acids via hepatic-intestine		1	(1)			3	(3)	
S1P degradation		1	(1)			1	(1)	
Transportation of bile aicds for kidney excretion		2	(2)			3	(3)	
DHS1P degradation		1	(1)			0		
Elimination of unsaturated fatty acids (PGE2) responsible for lipolysis inhibition		1	(1)			0		
phosphatidylcholine to phosphatidylethanolamine		1	(1)			0		
Sphingolipid biosynthesis		1	(1)			0		
SPH (SM) degradation		0				1	(0)	
Biosynthesis of cholesterol		0				10	(10)	
Biosynthesis of steroid hormone		0				1	(1)	
Biosynthesis of tryglycerides		0				1	(1)	
Biosynthesis of unsaturated fatty acids responsible for pro-inflammation		0				1	(1)	
Breakdown of phosphalipid to form unsaturated fatty acids		0				1	(1)	
Elimination of cholesterol (from cyculation back to liver for catabolism)		0				1	(1)	
Elimination of cholesterol via steoid hormone pathway		0				1	(1)	
Phosphatidylethanolamine to phosphatidylcholine		0				1	(1)	

**Figure 4:**
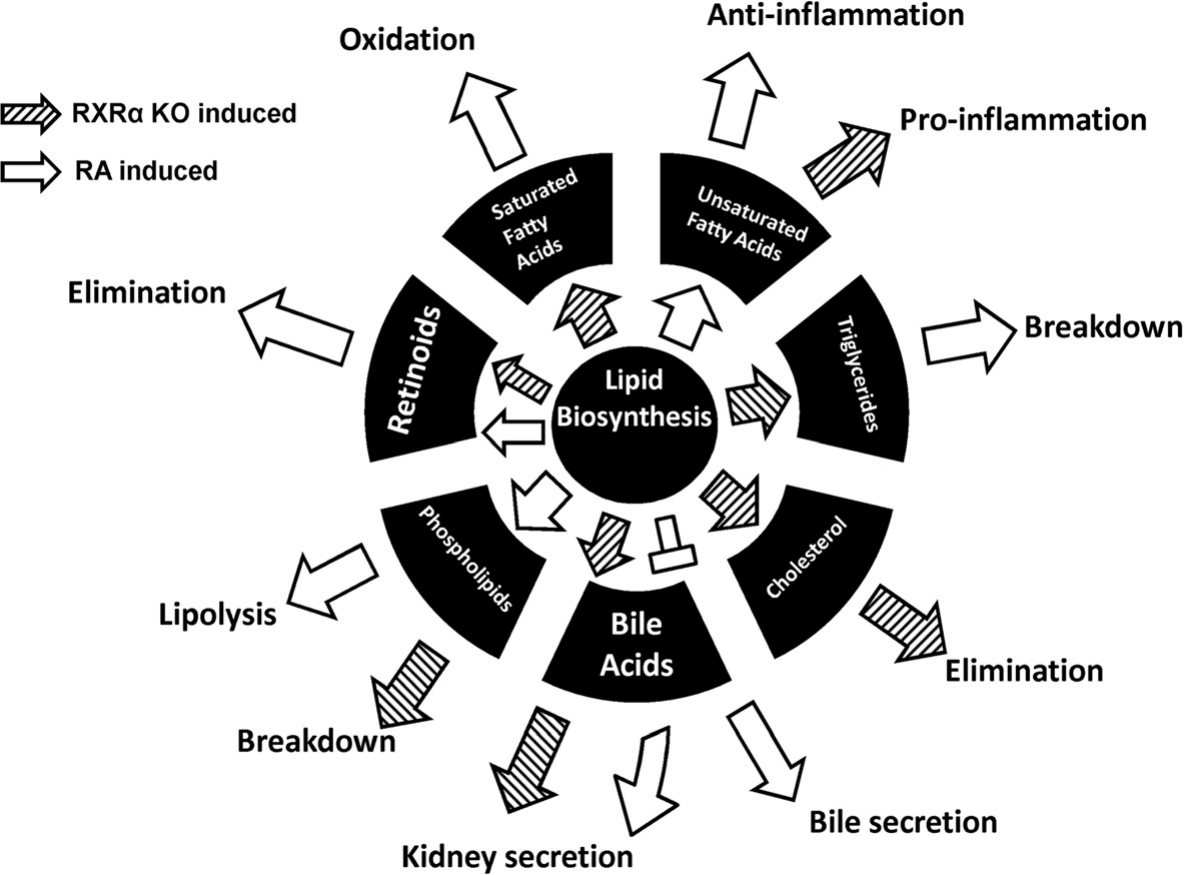
**Lipid synthesis and metabolism pathways regulated by RA treatment and RXRα deficiency.** The diagram represents the effect of RA treatment and RXRα deficiency on the expression of 114 lipid genes based on PCA analysis. Fifty-five genes were induced by RA and inhibited due to the lack of RXRα, and fifty-nine of them were induced because of RXRα deficiency and RA suppressing. Gene functions were obtained from the KEGG database and confirmed in PubMed gene database. The arrows inside the circle represent the lipid synthesis processes, and the outer arrows represent the lipid elimination processes. The “T” sign represents inhibition and all arrows represent inductions.

Taken together, ligand (RA) treatment and hepatic RXRα deficiency resulted in opposite effects. Figure 4 summarizes the effect of RA and hepatic RXRα deficiency on lipid homeostasis. RXRα deficiency tends to favor saturated fatty acids, triglyceride, cholesterol, and bile acids synthesis. In contrast, RA treatment leads to unsaturated fatty acids and phospholipid synthesis and lipolysis as well as triglyceride breakdown.

### Binding of RA/RXRα responsive genes by other nuclear receptors

**Figure 5:**
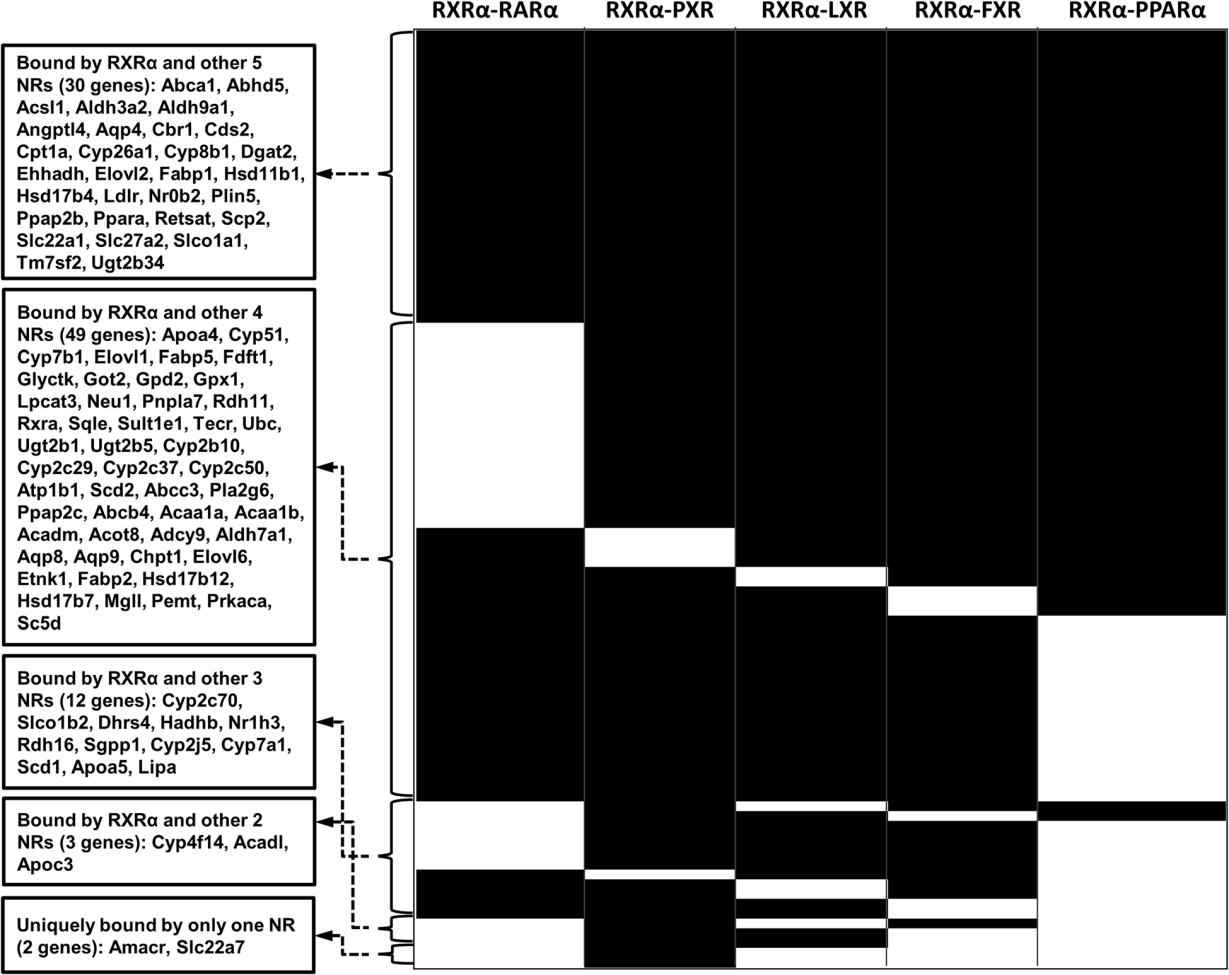
**Heat maps showing the binding profiles of RXRα/nuclear receptor on 96 RA-responsive and RXRα-dependent lipid genes.** RA responsive and RXRα-dependent genes (114) were analyzed by nuclear receptor binding. Among those 114 genes, 96 genes shown in black lines can be commonly regulated by RXRα and its partner. Some of the commonly regulated genes have nuclear receptor binding site at the same location.

Additional analysis was done to understand which other nuclear receptors may be involved in regulating the expression of these 114 RA/RXRα target genes, which have a role in lipid homeostasis. The binding data generated in the current study (RXRα and RARα) were compared with the binding data of PXR, LXR, FXR, and PPARα. Figure 5 shows overlapping genes with RXRα-heterodimers, as assessed by overlapping binding of RXRα and other nuclear receptors. The data were organized by the number of different nuclear receptors binding the genes. For example, motifs located in the Abca1, Abhd5, Acsl, and Aldh3a2 genes could be bound by RXRα and all five nuclear receptors. Peaks located in the Apoa4, Cyp51, Cyp7b1, and Elovl1 could be bound by RXRα and any 4 out of the 5 studied nuclear receptors (Figure 5). Some of the commonly regulated genes have nuclear receptor binding site at the same location. The data indicated extensive crosstalk among nuclear receptors in regulating the expression of those genes.

### Quantification of serum cholesterol, triglyceride, and bile acid levels

**Figure 6:**
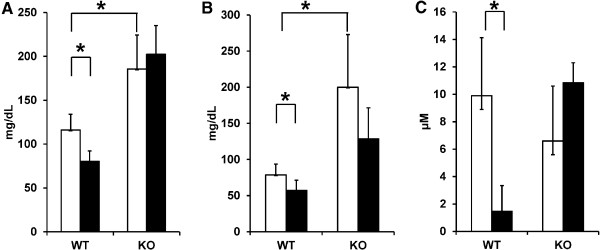
**Serum cholesterol, triglyceride, and bile acid levels in wild type and hepatocyte RXRα KO mice treated with and without RA.** Male wild type (WT) and hepatocyte RXRα KO (KO) mice were fed laboratory chow with (black bars) and without (white bars) all-trans RA (150 mg/kg diet) for 7 days (n = 6). Serum cholesterol **(A)**, triglyceride **(B)**, and bile acid **(C)** levels were quantified at the end of the treatment. * *p* < 0.01.

ChIP-Seq and RNA expression profiling indicate the role of RA in controlling lipid homeostasis in the liver. Serum cholesterol, triglyceride, and bile acid levels were quantified to test the genetic findings. The data showed that RA reduced serum cholesterol, triglyceride as well as bile acid levels in wild type mice (Figure 6). However, such effects were not found in hepatic RXRα KO mice. In addition, serum cholesterol and triglyceride, but not bile acid, levels were elevated due to RXRα deficiency. These biochemical findings confirm the role of RA in regulating lipid homeostasis in the liver.

## Discussion

This study establishes the role of nuclear receptors and RA in regulating lipid homeostasis in the liver. In addition, the mechanisms by which nuclear receptors and RA regulate lipid homeostasis were illustrated at the gene, pathway, and systemic levels. Furthermore, relationships between RXRα and PXR, LXR, FXR, as well as PPARα in regulating lipid homeostasis were analyzed. These nuclear receptors depend on RXRα to execute their functions and more than 85% of their bound genes overlap with those bound by RXRα. The nuclear receptor binding data were strengthened by profiling the expression patterns of 576 lipid genes, which showed that RA treatment and RXRα-deficiency had an opposite effect in regulating lipid homeostasis. Nuclear receptor binding data also illustrated extensive cross talk among the studied nuclear receptors. Thus, our novel in vivo data provided extensive evidence showing the role of RA in dictating lipid homeostasis in the liver.

It is intriguing that more than 85% of the PXR, LXR, FXR, and PPARα binding sites overlapped with those of RXRα. In contrast, only 43% of RARα binding sites overlapped with those of RXRα. It has been shown that RARα can form homodimers [22]. It is also possible that RARα may dimerize with RXRβ and RXRγ to exert its function. Although the percentage of overlapping bindings between RARα and RXRα is not as high as others, the number of genes that could be bound by RXRα/RARα (4554) is the highest, followed by RXRα/PPARα (3468), RXRα/FXR (2019), RXRα/LXR (988), and RXRα/PXR (666), implying the relatively extensive role of these nuclear receptors in regulating hepatic gene expression. It is important to further study the role of RARα and other RARs in the liver. By forming partners with other nuclear receptors, RXRα is a master regulator. Our data showed that more than 8000 hepatic genes were bound by RXRα, and 72% of them overlapped with the genes bound by RARα, PXR, LXR, FXR, or PPARα. The remaining 28% of RXRα binding sites might be bound by RXRα homodimer or the heterodimer of RXRα and VDR or CAR. Thus, the five nuclear receptors (RARα, PXR, LXR, FXR, or PPARα) analyzed in the current study account for almost three quarters of RXRα binding genes in the liver. Furthermore, nearly 50% of RXRα bindings overlapped with the bindings of PXR, LXR, FXR, and PPARα (Figure 1B). Hence, lipid regulation should be one of the major functions of RXRα.

Clustering and PCA showed that the genome-wide binding pattern of RARα is not similar to that of PPARα, LXR, PXR, and FXR. Biological function annotation also showed that RARα has some unique features including protein processing, protein localization, and RNA processing. However, the five studied nuclear receptors also demonstrated functional redundancy. For example, there are four pathways, including oxidation reduction, carboxylic acid catabolic process, organic acid catabolic process, and cofactor metabolic process, that can be regulated by more than four nuclear receptors. This finding suggests the importance of these four pathways in the liver, and the role of RARα in them.

Although PPARα, LXR, FXR, and PXR have extensive roles in regulating lipids, they also have specific roles in regulating different types of lipids. RXRα/PPARα prefers to bind to genes that participate in neutral lipids, glycerol ether, and organic ether as well as fatty acid metabolism processes. All of which are either fatty acid-derived products or precursors for the biosynthesis of fatty acids. Another pathway bound by RXRα/PPARα is the acylglycerol metabolic process, which is involved in triglyceride homeostasis. RXRα/LXR tends to regulate genes involved in sterol metabolism, which is consistent with its known role [23]. RXRα/FXR not only binds to the genes participating in steroid metabolism process, but also those involved in lipid transport and carbohydrate metabolism processes. RXRα/PXR binds to the genes involved in regulating the pyruvate metabolic process at the DNA binding level. Pyruvate is a key intersection for fatty acid, carbohydrate, and protein metabolisms. In addition, RXRα/PXR also regulates response to acute phase, inflammatory, and wounding, implying that PXR can be an excellent target for metabolism and inflammation-related health issues. Lastly, FXR binds to the genes involved in monosaccharide metabolism, which shows the intimate relationship between bile acid and glucose homeostasis [24].

All of the 114 genes that showed differential effects of RA treatment and RXRα deficiency are bounded by RXRα and RARα, PXR, LXR, FXR, and PPARα heterodimers. These findings indicate that those studied nuclear receptors retain RA response in vivo and the effect of RA is dependent upon those nuclear receptors. RA has a broad spectrum of effects including biosynthesis of retinoids, phospholipids, and unsaturated fatty acids. It also has a role in eliminating retinoids, oxidizing saturated fatty acids, and breaking down triglycerides. It seems that RA has extensive beneficial effects in maintaining the health of the liver. Specifically, RA induced the expression of Cyp2c37/38/50/54/70 and Cyp2j5. These genes encode enzymes involved in the generation of epoxyeicosatrienoic acids [25], which have anti-inflammatory effects [26]. In contrast, RXRα deficiency induces the gene expression of Cyp4f that is responsible for the generation of 20-hydroxyeicosatetraenoic acid, a pro-inflammation molecule [25]. In addition, RA increases mRNA levels of cbr1(carbonyl reductase 1), which is responsible for transforming prostaglandin E2 to prostaglandin F2α. Prostaglandin E2 and F2α have different effects in regulating lipid breakdown. Prostaglandin E2 is a lipolysis inhibitor [27]; whereas, prostaglandin F2α has not been shown to have the same effect. Thus, the induction of cbr1 gene expression could be a mechanism by which RA induces lipolysis. RA also induces expression levels of gene encoding proteins for phospholipid biosynthesis, but RXRα deficiency increases the expression of the genes that have a role in the degradation of phospholipids. This finding suggests the potential role of RA in maintaining the normal structure of the cell membrane. Formation of the monolayer of lipoprotein or lipid droplet is one of the major ways that phospholipids regulate lipid metabolism [28]. Phosphatidylethanolamine (PE) and phosphatidylcholine (PC) are two important phospholipids that show different effects on lipid metabolism in humans and rodents. Lower PC/PE ratio induces steatosis or even steatohepatitis in humans [29], however, PE has a greater effect than PC in reducing the cholesterol level in rodents [30]. Our data showed that RA induced the gene expression of ptdss1 (phosphatidylserine synthase 1), which converts PC to PE. Consistently, the ptdss1 gene expression is reduced due to hepatic RXRα deficiency. These gene expression levels and DNA binding data not only showed the underlying mechanism for RA in regulating liver gene expression, but also suggested the biochemical outcome.

## Conclusions

Taken together, the differentiation and morphogenetic effect of RA is well known. However, the current study provides a comprehensive analysis of the role of RA in lipid homeostasis. All-trans RA is the most abundant retinoid that can be easily detected in the liver. Thus, RA is likely to act as a regulator to control hepatic lipid metabolism. Since the effect of RA is broad, it is important to develop specific retinoids in order to target specific pathways. Such efforts may allow us to identify compounds that can be used to treat or prevent metabolic syndromes and other lipid-related health issues.

## Methods

### Materials and animal models

Male wild type mice (12 weeks old) and hepatocyte RXRα-deficient mice (KO) [10,11], which have the same genetic background of C57BL/6, were used. The RXRα KO mice were produced and characterized previously [10,11]. The LoxP sites were inserted into introns flanking the fourth exon of the RXRα gene covering the DNA binding domain, which is deleted after crossing the floxed RXRα allele against a transgenic line in which cre recombinase is expressed under the control of the albumin promoter. The mutant mice express a truncated protein that has the intact ligand binding domain, but lacks the DNA binding domain. Animal protocols and procedures were approved by the Institutional Animal Care and Use Committee (IACUC) at the University of Kansas Medical Center and the University of California, Davis.

All ChIP-grade antibodies except anti-RNA Pol II (Millipore, MA) were purchased from Santa Cruz Biotechnology, Inc. (Santa Cruz, CA). DNA purification kit was purchased from Qiagen Co. (Valencia, CA). All other ChIP-related reagents were obtained from Invitrogen Co. (Carlsbad, CA).

### Animal treatment

Retinoic acid (Sigma-Aldrich, MO) was given to wild type and hepatic RXRα-deficient mice at a dosage of 150 mg/kg diet for 7 days. As controls, mice were fed with normal diet.

### Chromatin immunoprecipitation (ChIP)

ChIP was performed according to our previously published study [31]. After fixation, the mouse livers were subjected to lysis with cell and nuclear lysis buffer. Sonication was used to fragment the chromatin, followed by precipitation with specified antibodies. The target DNA fragments were obtained by reverse crosslinking and purification. Antibodies against IgG and RNA Pol II were used as negative and positive controls, respectively.

### DNA library preparation and sequencing

By using the End-It DNA End Repair Kit (Illumina, Madison, WI), DNA fragments prepared from ChIP were ligated with specified adaptors and amplified, then size-selected (175-225 bp) on an agarose gel followed by sequencing (High-Seq 2000, Illumina, Madison, WI).

### Alignment, call peak, and annotation of ChIP-seq data

The target sequences were aligned to the mouse genome (http://hgdownload.cse.ucsc.edu/goldenPath/mm10/bigZips/) by Bowtie 0.12.7 [32] followed by peak-calling using MACS (version 1.4.1) [33]. The peaks were annotated using the database (NCBI37/mm9) by Peak Analyzer [34]. The background cut off standard was set to be 20 fold of the input signals [18]. The cut off distance from the transcription start site (TSS) was set to be 10 kb. Co-localization is defined as having at least 25% overlap in their peak widths.

### Microarray

Affymetrix 430 A_2 Chip (Santa Clara, CA) was used to determine the genome-wide mRNA expression levels. Microarray data were annotated using Affymetrix Expression Console (MAS5). The probe signal with *p* values less than 0.05 were used for further analysis.

### ChIP-seq data analysis

All data were treated with the same cut off criteria. The generated RXRα binding data were compared with the data for RARα, PXR, LXR, FXR, and PPARα. The principle component analysis (PCA) and cluster analysis package in SPSS program was used to analyze the global binding data. For both PCA and cluster analysis, called peaks were assigned the value of 1. Not called peaks were assigned the value of 0. Genes with overlapping binding sites of RXRα and each of RARα, PXR, LXR, FXR, and PPARα at the same location were functionally analyzed by the DAVID (http://david.abcc.ncifcrf.gov/) [35].

### Lipid homeostasis analysis based on mRNA expression

Genes (579) involved in regulating lipid homeostasis were extracted from the KEGG database (Kyoto Encyclopedia of Genes and Genomes, http://www.genome.jp/kegg/). The expression of those 579 genes were determined in wild type and liver RXRα KO mice treated with and without RA (n = 3) for 7 days. After multiple comparisons, only 30 and 36 out of all 579 lipid homeostasis genes showed significant change at the corrected p-value of 0.05 after RA treatment and RXRα knockout, respectively. Therefore, IBM SPSS PCA package was used to differentiate groups based on the global expression pattern of all 579 lipid homeostasis genes.

### Serum lipid assays

Triglyceride, cholesterol, and bile acids in the serum were assayed using a commercially available kit (Pointe Detroit, Michigan) that was modified to a 96-well format. Spectrophotometric analysis was conducted with a Bio-Tek microtiter plate reader (Bio-Tek, VT).

### Availability of supporting data

The microarray and ChIP-Seq data supporting the results of this article are available with accession numbers of GSE50028 and GSE46762, respectively, in the GEO repository (http://www.ncbi.nlm.nih.gov/geo/).

## Abbreviations

RA: Retinoic acid; ChIP: Chromatin immunoprecipitation; seq: Sequencing; RXRα: Retinoid x receptor alpha; RARα: Retinoic acid receptor alpha; PXR: Pregnane x receptor; LXR: Liver x receptor; FXR: Farnesoid x receptor; PPARα: Peroxisome proliferator-activated receptor alpha; DAVID: Database for annotation, visualization and integrated discovery; PCA: Principal component analysis; WT: Wild type; KO: Knockout; NR: Nuclear receptors; KEGG: Kyoto encyclopedia of Genes and genomes; PE: Phosphatidylethanolamine; PC: Phosphatidylcholine.

## Competing interests

The authors declare that they have no competing interests.

## Authors’ contributions

YH: Performed experiments, analyzed data, generated figures and tables as well as prepared manuscript. LG: Microarray experiments and analysis. YF: Sequence alignment and call peaks. QZ: Functional annotation. H-XL: Animal experiments. YL: Statistics analysis. GLG: FXR ChIP Seq data generation. LL-M: Microarray data generation. JF: Sequence alignment and call peaks. Y-JYW: Generated idea and supervised the all overall performance of the project. All authors read and approved the final manuscript.
